# Effect of sublingual fast-dissolving piroxicam premedication on postoperative pain experience in mandibular molars with non-vital pulp: a randomized double-blind controlled trial

**DOI:** 10.1186/s13005-024-00453-x

**Published:** 2024-09-21

**Authors:** Asmaa Fathelrahman Mohamed, Heba Ahmed El-Asfouri, Suzan Abdul Wanees Amin

**Affiliations:** Department of Endodontics, Faculty of Dentistry, Cairo University, 11 ElSaraya Str, ElManyal, Cairo, 11553 Egypt

**Keywords:** Mandibular molar, Piroxicam, Postoperative pain, Spontaneous, Stimulated

## Abstract

**Background:**

The aim of this trial was to evaluate the effect of a preoperative, single dose sublingual fast-dissolving piroxicam (20 mg) compared to placebo on postoperative pain at rest (POP), on biting (POPB) and on percussion (POPer) after single-visit endodontic treatment of asymptomatic mandibular molars with non-vital pulp.

**Methods:**

Seventy patients randomly received either piroxicam or placebo 1 h before treatment (*n* = 35). Patients recorded their pain (POP and POPB) level 6 h, 12 h, 24 h, 48 h, 72 h and 7 days postoperatively using an 11-point numerical rating scale; POPer was assessed after 7 days. Resuce-analgesic intake (RAI) and flare-up incidence (FUI) were recorded. Data were statistically analyzed.

**Results:**

Both groups had similar baseline characteristics (*P* > 0.05). Piroxicam showed less POP intensity and incidence than placebo at 6, 12 and 24 h, less POPB intensity and incidence at all timepoints, less POPer intensity and incidence and less RAI (*p* > 0.05), but similar FUI (*P* > 0.05). A significant rise in pain compared to baseline occurred with placebo from 6 to 72 h for POP and to 7 days with POPB (*p* > 0.05); such rise was not detected with piroxicam. POPB showed higher pain intensity than POP at all time points (*p* < 0.05). No swelling or adverse effects occured.

**Conclusions:**

A preoperative single dose of sublingual fast-dissolving piroxicam can be effective in reducing spontaneous pain up to 24 h, stimulated pain up to 7 days, and RAI incidence in asymptomatic mandibular molars with non-vital pulp; it can prevent rise in POP and POPB postoperatively. Stimulated postoperative pain can be more severe and longer lasting than spontaneous pain.

**Trial registration:**

Clinicaltrials.gov ID: NCT03998826 (2019).

**Supplementary Information:**

The online version contains supplementary material available at 10.1186/s13005-024-00453-x.

## Background

Postendodontic pain is a common complication after root canal treatment with an incidence ranging from 3% up to 64% [1–3]; 20% of which could experience severe pain [4]. According to its severity, duration and nature, it can affect patient’s wellbeing, particularly through interfering with function, and may predispose to more distressing persistent pain conditions [4–6]. Postoperative pain is more likely to increase, compared to baseline, in cases with no-to-mild mechanical allodynia [6]. Considering postoperative pain research, no trend in publications based on pulpal diagnosis was detected, with a recommendation for more focus on pulpal diagnoses more likely to experience postoperative pain [7]. Pain after non-surgical endodontic treatment is often inflammatory in nature which is mainly attributed microbiological factors; an infected root canal system is considered the main factor for flare-ups [7–9].

Among inflammatory mediators, prostaglandins have crucial functions in the pathogenesis of pulpal and periradicular diseases [9]. Use of systemic drugs, particularly a single oral dose of non-steroidal anti-inflammatory (NSAIDs) as premedication, to reduce the inflammatory reactions can be effective in reducing postendodontic pain [10]. The main mechanism of action of most NSAIDs is prostaglandins’ inhibition acting primarily through the inhibition of cyclooxygenase (COX) enzymes 1 and 2. Inhibiting COX-2, blocks prostaglandin formation and ultimately prevents inflammation and sensitization of the peripheral nociceptors; of all NSAIDs for endodontic use, piroxicam is the longest-acting [10, 11].

Piroxicam is a potent, non-selective, reversible COX-1 and COX-2 inhibitor of the oxicam class that has long-acting, anti-inflammatory action with minimal side effects e.g. gastric intolerance [11]. Oral piroxicam is available in different forms including capsules, dispersible tablets, to be dissolved in water before intake, or fast-dissolving tablets (FDTs) administrated sublingually to enhance patient acceptability [12]. Few studies have assessed the effect of preoperative oral piroxicam administration on the postendodontic pain, up to a maximum of 72 h [13–16]. Some studies demonstrated efficacy for 20 mg piroxicam up to 24 h [14, 15], and 72 h [16], while another showed efficacy for 40 mg till 48 h [13]. In two studies, however, no placebo was administered in the control group [13, 16], and, in one study, no randomization was implemented [14]. Previous studies included patients with symptomatic teeth with vital pulp [13, 15, 16], or teeth with vital and non-vital pulp [14]. The purpose of this study, thus, was to assess the effect of a preoperative, single dose of oral, sublingual piroxicam tablets (20 mg) on postoperative pain experience regarding pain at rest (POP) compared to pain on biting (POPB), pain on percussion (POPer) and resuce analgesic intake (RAI) and flare-up incidence (FUI) over a 7-day duration in patients with asymptomatic mandibular molars having non-vital pulps treated in a single visit. The null hypothesis was that there is no difference in postoperative pain experience regarding POP, POPB, POPer, RAI and FUI over 7 days after administration of preoperative, single dose of oral, sublingual piroxicam tablets (20 mg) compared to placebo.

## Methods

### Study design, setting and sampling

The protocol of this randomized, parallel-arm, double-blind, placebo-controlled clinical trial (allocation ratio 1:1) and the informed consent format were approved by the Research Ethics Committee (19/7/16), Faculty of Dentistry, Cairo University. The study protocol was registered on www.clinicaltrials.gov (Clinicaltrials.gov ID: NCT03998826). This trial was reported according to the CONSORT 2010 guidelines (Additional file 1). A written informed consent was obtained from each patient after explaining treatment steps, benefits and possible risks. The study took place in the outpatient clinic of the Department of Endodontics, Faculty of Dentistry and clinical procedures were performed by a postgraduate student in the duration from December 2020 to July 2021.

### Sample size calculation

Based on data from a trial including teeth with asymptomatic non-vital pulp, a type I error of 0.05, and statistical power of 80%, the required sample size was 62 participants, 31 per group, to detect a minimal clinically-important difference of 34% in postendodontic pain incidence with an expected incidence of 57% in the control group [17]. The number was increased to 70 participants to compensate for dropouts. Sample size calculation was performed using PS: Power and Sample Size Calculation Version 3.1.2 (http://biostat.mc.vanderbilt.edu/wiki/Main/PowerSampleSize).

### Eligibility criteria

Each included participant had a mandibular molar (First or second) with asymptomatic, non-vital pulp, aged between 18–50 years old, was in good health [American Society of Anesthesiologists (ASA) Class I or II] and took no drug that good affect pain perception (e.g. analgesics, anti-inflammatory drugs) for at least a week and no antibiotics for at least 3 months. Exclusion criteria were as follows: patients who had allergies or sensitivity to piroxicam or any other medicament/material used in the study, pregnant or nursing females, patients with a history of active peptic ulcer within the preceding 12 months, bleeding problems, or anticoagulant use, patients with periapical abscess, sinus tract and/or previous endodontic treatment in the target tooth, and/or patients unable to provide an informed consent.

### Diagnosis

Diagnosis was based on the patient’s chief complaint, history taking and clinical and radiographic examination. The included patients had a diagnosis of asymptomatic mandibular molars with nonvital pulps, associated or not with radiographic evidence of apical periodontitis (AP) (≤ 5 mm in diameter). Patient were asked to rate their pain at rest (PreOP) on an 11-point numerical rating scale (NRS) pre-operatively. Patients were also asked to record upon biting on a wooden tongue blade (PreB). The operator performed a percussion test and pain upon percussion (PrePer) was recorded. All patients scored zero pain preoperatively. The NRS was divided into 4 categories, to enhance clinical interpretability, so that zero score indicated “no pain”, 1–3 indicated “mild pain”, 4–6 indicated “moderate pain” and 7–10 indicated “severe pain”, 10 indicated “The worst pain”. Patients had no sensitivity response to a cold pulp-sensibility test (Endo-Ice spray, Henry Schein, Germany), no tenderness to percussion or palpation. Each tooth showed normal periodontal probing and mobility. Diagnosis was confirmed by lack of bleeding on access preparation. Patients, also, completed the Modified Dental Anxiety Scale (MDAS) to rate their level of anxiety; data were dichotomized based on the average MDAS scores into < 12 (Calm) and ≥ 12 (Anxious) [18]. The patients were, also, asked about the time of last food intake so that eating within < 8 h was designated as “Ate”, and ≥ 8 h as”Fast”.

### Blinding and Randomization

Placebo tablets were prepared by a licensed pharmacist identical to piroxicam ones so that both the patient and the operator were unaware of the assigned group until the trial’s end; both tablet types were packed in similar, opaque containers. Randomization was done using the permuted-block method with variable-sized blocks of 4 and 6 and the sequence was generated using computer software (Excel; Microsoft, Redmond, WA). Allocation concealment was done through using sequentially-numbered, opaque, sealed containers. Randomization was done by an investigator not involved in the enrollment of patients into the study.

### Endodontic procedures

The patients randomly received either 20 mg sublingual fast-dissolving tablet (FDT) of piroxicam or placebo one hour before anesthetic administration. Each patient, then, received an inferior alveolar nerve block using a standard dental aspiration syringe with 27-gauge needle. The anesthetic solution was 1.8 ml of 2% mepivacaine hydrochloride with levonordefrin 1:20,000 (Mepcaine-L, Alexandria Company for pharmaceuticals and Chemical Industries, Alexandria, A.R.E). Endodontic access was performed using a size 4 round bur and an endodontic access bur (Endo-Z Bur, Dentsply Sirona, Ballaigues, Switzerland). Each tooth was isolated using rubber dam. Working length was determined using an apex locator (Root ZX mini, J Morita Corp, Kyoto, Japan) and radiographically confirmed as 0.5–1 mm shorter from the radiographic apex. Root canal instrumentation was, then, done using a rotary nickel-titanium system (ProTaper Next, Dentsply, Maillefer, Ballaigues, Switzerland). Narrow and curved canals were prepared up to X2 instrument (25/0.06). Large canals were prepared up to X3 (30/0.07) or X4 instruments (40/0.06). Irrigation was done using 2 mL of 2.5% sodium hypochlorite between every two consecutive instruments. Final flush was done using 2 ml of 17% EDTA followed by 5 ml distilled water. Canals were, then, dried with paper points (META BIOMED CO., LTD, Korea) and were obturated using modified single-cone technique and epoxy resin sealer (Adseal, Meta Biomed C0., Ltd., Chungbuuk, Korea). Access cavity was, then, sealed with a temporary filling (MD-TEMP, Meta Biomed C0., Ltd., Chungbuuk, Korea).

### Postoperative pain assessment

Each patient received a pain diary to record the intensity of pain felt after 6, 12, 24, 48, 72 h and 7 days at rest (POP) and on biting (POPB). Pain assessment was done using NRS; patients were trained to use it before recording pain. The operator phoned patients at each time point to check on them and to be remind them of recording their pain. If pain persisted, patients were instructed to take rescue analgesic (200 mg ibuprofen; Brufen, Abbott Laboratories) was prescribed; patients recorded their rescue analgesic intake (RAI). Incidence of flare-up, defined as severe pain and/or swelling that interefered with the patient’s lifestyle, [1, 9] was also recorded. After 7 days, patients submitted their pain diaries to the operator who performed percussion test and pain was recorded (POPer). Patients were referred to the restorative department for final restoration.

### Statistical analysis

Data were coded to allow statistician blinding and statistically analyzed using the Statistical Package for Social Sciences version 25 (SPSS Inc., IBM Corporation, NY, USA). Descriptive analysis for all variables was performed. Data were explored for normality using Kolmogorov–Smirnov test and Shapiro–Wilk test and postendodontic pain intensity demonstrated non-normal distribution (*p* < 0.05). Comparisons for continuous normally-distributed variables were done using Student’s *t*-test. Non-normally-distributed continuous variables were compared by Mann–Whitney U test. Related-group comparisons were done using Friedman’s test followed by Wilcoxon’s sign rank test for multiple comparisons. Multiple linear (MLR) was used to assess the association between continuous outcome variables and independent variables which included: Group (*PRX/PLC*), sex (*Male/Female*), Molar (*First/Second*), periapical lesion size (> *2-5 mm/0-2 mm*), MDAS score, Anxiety (*Anxious/Calm*) and food intake (*Fast/Ate*). For categorical variables, differences were analyzed using Pearson’s Chi square (*X*^2^) test and Fisher’s exact test when appropriate. The relative risk (RR) and its 95% confidence intervals (CI) for the risk of pain, FUI, and RAI were estimated. Statistical significance (α) was set at 0.05.

## Results

**Fig. 1:**
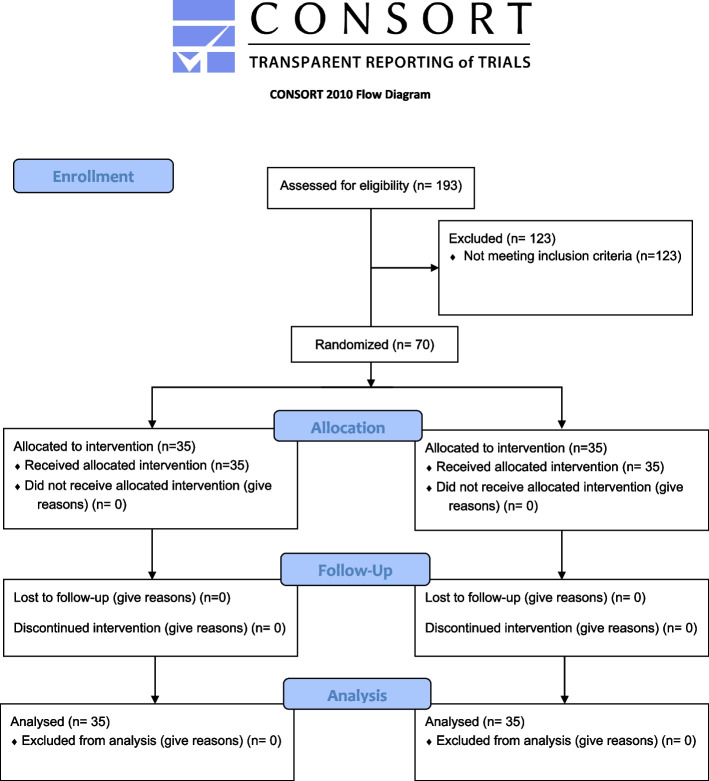
The Consolidated Standards of Reporting Trials flow diagram of the trial

Of 193 patients assessed for eligibility, 70 (43 females, 27 males) were randomized and analyzed (Fig. 1). Patients’ age ranged from 18 to 60 years. The study included fifty-one (72.9%) first molars and 19 (27.1%) second molars. Periapical lesion size in 41 (58.6%) molars was 0-2 mm and in 29 (41.4%) was > 2-5 mm. There was no difference between groups regarding age, sex distribution, molar-type distribution, periapical lesion size, MDAS score, and last food intake (*p* > 0.05, Table 1). An intention-to-treat analysis was adopted.

**Table 1:** Baseline characteristics of study participants in the piroxicam (PRX) and the placebo (PLC) groups

Variable	Categories	PRX (*n* = 35)	PLC (*n* = 35)	*P*-Value
**Age**	***Mean*** ± ***SD***	32.66 (10.30)	33.4 (9.95)	0.76
	***Median***	33	32	
	***(Min- Max)***	(19—60)	(18—60)	
**Sex**	***Males [n (%)]***	10 (28.6)	17 (48.6)	0.086
	***Females [n (%)]***	25 (71.4)	18 (51.4)	
**Molar Type**	***1st [n (%)]***	26 (74.3)	25 (71.4)	0.788
	***2nd [n (%)]***	9 (25.7)	10 (28.6)	
**Number of canals**	***3 [n (%)]***	30 (85.7)	25 (82.9)	0.743
	***4 [n (%)]***	5 (14.3)	10 (17.1)	
**Periapical lesion size**	***0-2 mm [n (%)]***	20 (57.1)	21 (60.0)	0.143
	** > *2-5 mm [n (%)]***	15 (42.9)	14 (40.0)	
**Last food intake**	** ≥ *8 h (Fast) [n (%)]***	14 (40.0)	13 (37.1)	0.806
	** < *8 h (Ate) [n (%)]***	21 (60.0)	22 (62.9)	
**MDAS**	** ≥ *12 (Anxious) [n (%)]*** ** < *12 (Calm) [n (%)]***	22 (62.9) 13 (37.1)	16 (45.7) 19 (54.3)	0.150

*Max* Maximum, *MDAS* Modified Dental Anxiety Scale, *Min* Minimum, *SD*, Standard deviation

The incidence of POP, POPB and POPer at the different pain categories (no, mild, moderate and severe) is shown in Table 2. Overall POP incidence was 31.4% at 6 h, 30.0% at 12 h, 28.6% at 24 h, 17.1% at 48 h, 11.4% at 72 h, and 8.6% at 7 days; fewer patients had POP, regardless of severity, with piroxicam than placebo at 6 h, 12 h and 24 h (*p* < 0.05). Overall POPB incidence was 31.4% at 6 h, 30.0% at 12 h, 30.0% at 24 h, 27.1% at 48 h, 24.3% at 72 h, and 18.6% at 7 days; fewer patients had POPB with piroxicam than placebo at all timepoints (*p* < 0.05).

**Table 2:** Incidence and percentage of pre- and postoperative pain at rest, on biting and on percussion

Timepoint	Pain category	PRX *(n* = *35)* *n (%)*	PLC *(n* = *35)* *n (%)*	Timepoint	Pain category	PRX *(n* = *35)* *n (%)*	PLC *(n* = *35)* *n (%)*
POP				**POPB**			
PreOP	***No pain***	35 (100)	35 (100)	**PreB**	***No pain***	35 (100)	35 (100)
POP6h	***No pain*** ***Mild*** ***Moderate*** ***Severe***	31 (88.6) 1 (2.9) 2 (5.6) 1 (2.9)	17 (48.6) 7 (20.0) 6 (17.1) 5 (14.3)	**POPB6h**	***No pain*** ***Mild*** ***Moderate*** ***Severe***	30 (85.8) 2 (5.6) 1 (2.9) 2 (5.6)	18 (51.4) 2 (5.6) 7 (20.0) 8 (22.9)
POP12h	***No pain*** ***Mild*** ***Moderate*** ***Severe***	32 (91.4) 0 (0.0) 1 (2.9) 2 (5.6)	17 (48.6) 9 (25,7) 4 (11.4) 5 (14.3)	**POPB12h**	***No pain*** ***Mild*** ***Moderate*** ***Severe***	31 (88.6) 1 (2.9) 0 (0.0) 3 (8.6)	18 (51.4) 2 (5.6) 8 (22.9) 7 (20.0)
POP24h	***No pain*** ***Mild*** ***Moderate*** ***Severe***	32 (91.4) 0 (0.0) 2 (5.6) 1 (2.9)	18 (51.4) 10 (28.6) 3 (8.6) 4 (11.4)	**POPB24h**	***No pain*** ***Mild*** ***Moderate*** ***Severe***	31 (88.6) 1 (2.9) 1 (2.9) 2 (5.6)	18 (51.4) 3 (8.6) 9 (25.7) 5 (14.3)
POP48h	***No pain*** ***Mild*** ***Moderate*** ***Severe***	32 (91.4) 1 (2.9) 1 (2.9) 1 (2.9)	26 (74.3) 4 (11.4) 4 (11.4) 1 (2.9)	**POPB48h**	***No pain*** ***Mild*** ***Moderate*** ***Severe***	32 (91.4) 0 (0.0) 2 (5.6) 1 (2.9)	19 (54.3) 6 (17.1) 8 (22.9) 2 (5.6)
POP72h	***No pain*** ***Mild*** ***Moderate*** ***Severe***	32 (91.4) 2 (5.6) 0 (0.0) 1 (2.9)	30 (85.8) 2 (5.6) 3 (8.6) 0 (0.0)	**POPB72h**	***No pain*** ***Mild*** ***Moderate*** ***Severe***	32 (91.4) 0 (0.0) 2 (5.6) 1 (2.9)	21 (60.0) 10 (28.6) 4 (11.4) 0 (0.0)
POP7d	***No pain*** ***Mild***	33 (94.3) 2 (5.7)	31 (88.6) 4 (11.4)	**POPB7d**	***No pain*** ***Mild*** ***Modertate***	32 (91.4) 2 (5.6) 1 (2.9)	25 (71.4) 8 (22.9) 2 (5.6)
PrePer	***No pain***	35 (100)	35 (100)	**POPer7d**	***No pain*** ***Mild*** ***Modertate***	28 (80.0) 4 (11.4) 3 (8.6)	17 (48.6) 11 (31.4) 7 (20.0)

*PLC* Placebo group, *POP* Postoperative pain at rest, *POPB* Postoperative pain on biting, *POPer* Postoperative pain on percussion, *PreB* Preoperative pain on biting, *PreOP* Preoperative pain at rest, *PrePer* Preoperative pain on percussion, *PRX* Piroxicam group

**Fig. 2:**
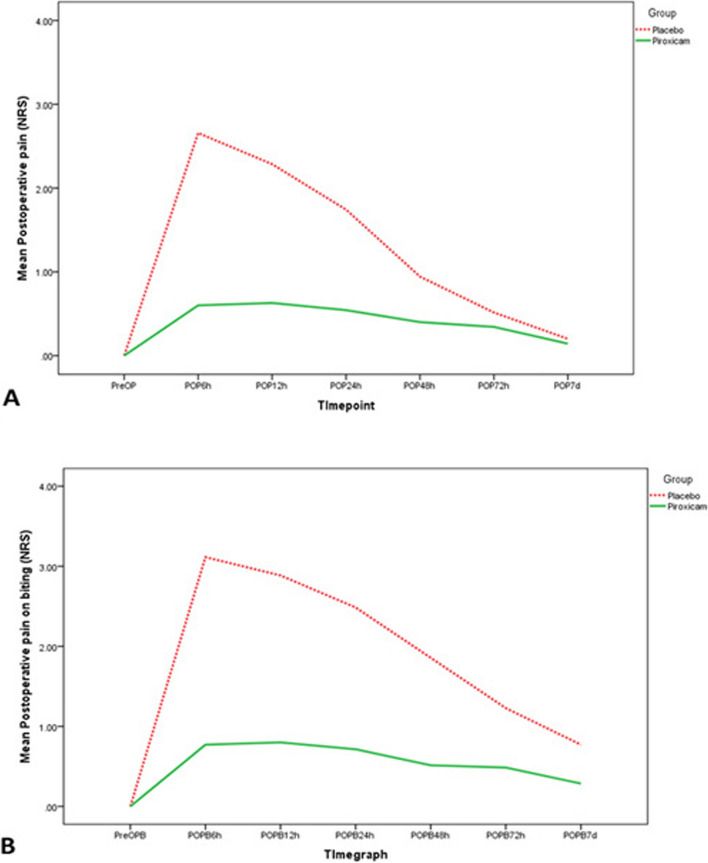
Pain intensity over time. Pain intensity (**A**) at rest (POP), and on biting (POPB) over time for the piroxicam and the placebo groups at the different timpoints (preoperatively, 6 h, 12 h, 24 h, 48 h, 72 h and 7d postoperatively)

Piroxicam showed significantly less intensity of POP than placebo at 6 h, 12 h, 24 h, of POPB at all timepoints and of POPer at 7d (*p* < 0.05, Table 3). Results showed significantly higher pain intensity of POPB than POP at all time points (*p* < 0.05). Change in POP and POPB intensity over time for both groups is shown in Fig. 2A and B respectively. A significant rise in POP compared to PreOP occurred at 6 h, 12 h, 24 h, 48 h and 72 h with placebo and that rise occurred up to 7 days with POPB; a significant gradual decline in pain level, however, occurred between every two successive postoperative timpoints (*p* < 0.05, Table 3 and Fig. 2A and B respectively). No such increase in POP occurred at any postoperative timepoint relative to preoperative pain with piroxicam (*p* > 0.05, Table 3 and Fig. 2A) and a rise only at 6 h in POPB (*p* < 0.05, Table 3 and Fig. 2B). POPer at 7d was significantly higher than PrePer occurred in both groups (*p* < 0.05, Table 3). MLR findings of the best-fit models revealed that POP intensity at 6 h, 12 h and 24 h, POPB intensity at 6 h, 12 h and 24 h, and 48 h, and POPer intensity at 7d were associated with *Group* where PRX was associated with less pain intensity (*p* < 0.05, Table 4).

**Table 3:** Pain intensity of postoperative pain at rest (POP), on biting (POPB) and on percussion (POPer)

Time point	PRX (*n* = *35*)				PLC (*n* = *35*)				*p*-value†
	***Med***	***Min***	***Max***	***Mean (SD)***	***Med***	***Min***	***Max***	***Mean (SD)***	
***Postoperative pain at rest (POP)***									
*** PreOP***	0^Aa^	0	0	0.00 (0.00)	0^Bg^	0	0	0.00 (0.00)	1.000
*** POP6h***	0^Aa^	0	10	0.6 (1.97)	2^Aa^	0	10	2.66 (3.26)	< 0.001*
*** POP12h***	0^Aa^	0	10	0.63 (2.17)	2^Ab^	0	10	2.29 (2.91)	< 0.001*
*** POP24h***	0^Aa^	0	9	0.54 (1.90)	0^Ac^	0	8	1.74 (2.44)	0.001*
*** POP48h***	0^Aa^	0	7	0.4 (1.42)	0^Ad^	0	7	0.94 (1.88)	0.071
*** POP72h***	0^Aa^	0	7	0.34 (1.30)	0^Ae^	0	5	0.51 (1.34)	0.450
*** POP7d***	0^Aa^	0	3	0.14 (0.60)	0^Bf^	0	2	0.20 (0.58)	0.432
***p*-value ‡**	0.014*				< 0.001*				
***Postoperative pain on biting (POPB)***									
*** PreB***	0^Bb^	0	0	0.00 (0.00)	0^Bg^	0	0	0.00 (0.00)	1.000
*** POPB6h***	0 ^Aa^	0	10	0.77 (2.25)	0^Aa^	0	10	3.11 (3.60)	0.002*
*** POPB12h***	0 ^Ba^	0	10	0.80 (2.49)	0^Ab^	0	10	2.89 (3.30)	0.002*
*** POPB24h***	0 ^Ba^	0	9	0.71 (2.22)	0^Ac^	0	8	2.49 (2.96)	0.002*
*** POPB48h***	0 ^Ba^	0	7	0.51 (1.72)	0^Ad^	0	7	1.86 (2.35)	0.001*
*** POPB72h***	0 ^Ba^	0	7	0.49 (1.63)	0^Ae^	0	6	1.23 (1.77)	0.006*
*** POPB7d***	0 ^Ba^	0	4	0.29 (0.96)	0^Af^	0	5	0.77 (1.35)	0.046*
***p*-value ‡**	0.001*				< 0.001*				
***Postoperative pain on percussion (POPer)***									
*** PrePer***	0^B^	0	0	0.00 (0.00)	0^B^	0	0	0.00 (0.00)	1.000
*** POPer7d***	0^A^	0	6	0.66 (1.51)	2^A^	0	5	1.74 (1.87)	0.004*
***p*-value β**	0.016*				< 0.001*				
***Number of analgesic tablets***									
	0	0	5	0.433 (1.33)	0	0	6	1.11 (1.73)	0.019*

^†^ Mann–Whitney test comparing two independent groups in a row

‡Friedmann’s test comparing different time-points within each group in a column; different upper-case letters within each column represent a significant difference of each postoperative time-point from the preoperative time-point within each group (Wilcoxon’s sign rank test); different lower-case letters within each column represent a significant difference of each time-point from the one preceding it within each group (Wilcoxon’s sign rank test); β Wilcoxon’s sign rank test;

* statistical significance at *p* ≤ 0.05. *Max* maximum, *Med* median, *Min* minimum, *PLC* Placebo group, *PRX* Piroxicam group, *SD* standard deviation

**Table 4:** Effect of variables on postoperative pain intensity at rest, on biting and on percussion

Timepoints	Variables (Reference)	B	S.E	β	*p*-value
***Postoperative pain at rest (POP)***					
**6 h**	**Group (*PLC*)**	-1.850	0.662	-0.325	0.007*
	**MDAS score**	-0.066	0.062	-0.123	0.292
	**Periapical lesion size (*0-2 mm)***	-0.543	0.654	-0.094	0.410
	**Food intake (*Ate*)**	-0.925	0.663	-0.158	0.168
**12 h**	**Group (*PLC*)**	-1.620	0.612	-0.304	0.010*
	**Food intake (*Ate*)**	-0.867	0.628	-0.159	0.172
	**Molar (1st)**	0.449	0.688	0.075	0.516
**24 h**	**Group (*PLC*)**	-1.163	0.516	-0.260	0.027*
	**Food intake (*Ate*)**	-1.037	0.530	-0.226	0.054
	**Molar (1st)**	0.243	0.580	0.048	0.676
***Postoperative pain on biting (POPB)***					
**6 h**	**Group (*PLC*)**	-2.288	0.718	-0.359	0.002*
	**Food intake (*Ate*)**	-1.100	0.739	-0.168	0.142
	**Molar (1st)**	0.022	0.822	0.003	0.978
	**Periapical lesion size (*0-2 mm)***	-0.791	0.743	-0.122	0.291
**12 h**	**Group (*PLC*)**	-2.023	0.696	-0.330	0.005*
	**Food intake (*Ate*)**	-1.118	0.717	-0.178	0.124
	**Molar (1st)**	0.321	0.797	0.047	0.689
	**Periapical lesion size (*0–2)***	-0.756	0.721	-0.121	0.298
**24 h**	**Group (*PLC*)**	-1.723	0.624	-0.316	0.008*
	**Food intake (*Ate*)**	-1.085	0.643	-0.194	0.096
	**Molar (*1st*)**	0.054	0.714	0.009	0.940
	**Periapical lesion size (*0-2 mm)***	-0.569	0.646	-0.103	0.382
**48 h**	**Group (*PLC*)**	-1.299	0.498	-0.303	0.011*
	**Food intake (*Ate*)**	-0.916	0.506	-0.208	0.075
	**Molar (*1st*)**	0.239	0.565	-0.050	0.674
	**Anxiety (*Calm*)**	-0.066	0.513	-0.015	0.899
***Postoperative pain on percussion (POPer)***					
**7d**	**Group (*PLC*)**	-1.103	0.412	-0.313	0.009*
	**Food intake (*Ate*)**	-0.610	0.418	-0.169	0.149
	**Anxiety (*Calm*)**	0.203	0.414	0.057	0.626

^*^Statistical significance (*p* < 0.05); B, unstandardized coefficient**;** β, standardized coefficient;. 1st, first mandibular molar; 2nd, second mandibular molar; *PLC* Placebo group, *PRX* piroxicam group, *S.E.* Standard error

Six of the 70 patients (8.6%) experienced flare-up of which 2 of 35 patients occurred with piroxicam (5.7%) and 4 of 35 patients (11.4%) with placebo (*p* = 0.393). No patient experienced swelling.

Seventeen of the 70 patients (24.3%) required RAI of which 4 of 35 patients (11.4%) with piroxicam and 13 of 35 (37.1%) with placebo (*p* = 0.012). Patients in the piroxicam group took less number of rescue analgesic tablets than in the placebo group (*p* = 0.019, Table 3). No adverse effects were detected in both groups.

## Discussion

Premedication with the 20-40 mg oral piroxicam has shown efficacy in preventing spontaneous POP particularly with symptomatic irreversible pulpitis [13–16]; the efficacy of 20 mg sublingual piroxicam FDT on spontaneous compared to stimulated postoperative pain as well as RAI and FUI was, yet, to be investigated in cases of non-vital pulp which can be more likely to experience postoperative pain rise.

Asymptomatic teeth with non-vital pulp were included for being more susceptible to pain rise after treatment [6, 7, 19]. Also, preoperative pain strongly predicts postoperative pain [3, 4, 20]; including asymptomatic teeth could, thus, avoid its confounding effect allowing more effective differentiation of the effect of piroxicam administration on different postoperative pain types. Mandibular molars were associated with more postoperative pain [3, 6]. This study, thus, represented a ‘worst case scenario’ for postoperative pain experience. A standardized protocol was implemented to minimize the effect of possible intraoperative confounders [21, 22]. Moderate certainty exists that single-visit treatment with AP can provide better radiographic outcome than multiple-visit treatment [23].

Various tools have been used for pain assessment of which NRS shows higher compliance rates, responsiveness, with high reliability and validity [24]. Patients were trained to use NRS to reduce measurement bias [21]. A 7-day trial duration, compared to a maximum of 3 days in previous studies [13–16], can be sufficient to allow the manifestation of the preemptive effects of piroxicam, the longest-acting NSAID used in endodontics [10] if they exist. Most studies assess spontaneous rather than stimulated postoperative pain [6, 10, 19]. Stimulated pain can be assessed on chewing, biting and/or percussion; a bite test can better simulate patient’s pain on chewing than percussion test [6, 19].

This study was a double-blind, randomized placebo-controlled, clinical trial with an allocation ratio of 1:1 where block randomization and blinding should reduce allocation bias risk, performance and ascertainment bias respectively [21]. In the present study, baseline characteristics were balanced between the two groups.

Compared to previous studies including asymptomatic teeth with necrotic pulp, POP characteristics (Intensity and/or incidence) in their control groups were either comparable to [25], higher than [17, 26] or lower than [27, 28] the findings of the control group in this study across corresponding timepoints. Discrepancies could be rendered to variations in population’s criteria, e.g. sex, tooth type, periapical radiolucency, in treatment protocols implemented e.g. number of visits, instrumentation kinematics, occlusal reduction, and/or pain scales [17, 26–28].

Piroxicam showed less severity and incidence of POP within the first 24 h, POPB for the first week, and POPer at day 7, and less RAI incidence. So the null hypothesis was rejected. This was in partial agreement with previous studies [13–16] where piroxicam showed efficacy for 24 h [14, 15], 48 h [13], or 72 h [16]. Differences in efficacy duration could be rendered to variations in patients’ criteria, in treatment protocols e.g. dose [13] and administration time, use of placebo [13, 16], endodontic procedures [14, 15], in pain type assessed, and/or methodological differences e.g. follow-up duration and randomization [14]. Most previous studies included patients with symptomatic irreversible pulpitis [13, 15, 16], and posterior teeth [13–15].

Apparent differences could be detected for the trend over time for postoperative pain (POP and POPB) between piroxicam and placebo groups where a significant rise occurred with placebo compared to piroxicam. This was in disagreement to the results of previous relevant studies assessing piroxicam efficacy [13–15]; all included symptomatic teeth so only a decline in pain incidence and/or intensity over time from the preoperative pain levels was reported. The findings of this study, particularly of the control group, are in agreement with previous studies with asymptomatic teeth, where a rise in pain levels can occur in the first 24 h [2, 6, 17, 25–28]. The extended piroxicam efficacy up to 7 days with POPB compared to 24 h with POP could be attributed to the higher severity of POPB and for a longer duration than POP (Table 3, Fig. 2A and B) as well as possible preemptive analgesic effects [18].

Piroxicam, an enolic-acid derivative, is a non-selective NSAID with the main mechanism of action being the inhibition of the cyclooxygenase enzymes, resulting in reduced prostaglandin synthesis [11]. Piroxicam may, also, inhibit activation and aggregation of neutrophils, hence, implying additional mechanisms of action including decreasing proinflammatory cytokine levels [11]. Piroxicam inhibits thromboxane synthesis in platelets, thus, inhibits the secondary phase of platelet aggregation; given the role of platelets in the inflammatory process, this action may contribute to the efficacy of piroxicam [29]. Piroxicam has the longest half-life (50 h) of all NSAIDs due to a low systemic clearance rate with 99% protein binding; time to peak plasma drug concentration after a single dose is 3-5 h [11]; taken together, this could explain its ability to inhibit pain rise and/or flare-up which reached its maximum at 6 h in this study and maintain such pain inhibition throughout the study duration. Pretreatment analgesia before root canal treatment may decrease the establishment of peripheral and central sensitization which has the potential to reduce postoperative pain and rescue analgesic intake [18].

Few studies have assessed stimulated postendodontic over time [6, 20]. Stimulated pain is usually more severe and lasts longer than spontaneous pain [6, 19, 30]; this was supported by the findings of this study where POPB showed higher severity and longer duration than POP (Table 3, Fig. 2B). Henry et al. (2001), however, showed similar spontaneous and stimulated pain values [18]. In this study, POPB levels were within the mild range and incidence range between 28 and 49% throughout 7 days in the control group. Lower stimulated pain levels, however, were reported by Jang et al. (2021), probably due to their pain assessment after the last visit of 2–3 visits and performing occlusal reduction [6]. Henry et al. (2001) reported higher incidence range (41% to 91%) in patients with ‘symptomatic’ teeth and assessing ‘percussion’ pain [19, 20].

Within the limits of this study, premedication seemed to be the most prominent factor associated with POP and POPB overwhelming the effect of other factors (Table 4). Food intake tended to affect POP at 24 h and POPB at 24 h and 48 h at 10% level of significance where *Fasting* patients had less pain intensity than those who *Ate* (Table 4). This could be due to the presence of food whichincreases the mean time to reach maximum plasma concentration compared to the fasting state [31, 32].

The findings of the present study could be limited by the relatively small sample size, which could affect the precision of the estimates. Only mandibular molars were included which could affect the generalizability to other teeth types, however, results of this study could simulate the worst case scenario. Performing randomization, patient- and operator-blinding together with absence of loss to follow up could be considered strengths in this study improving its internal validity. The choice of patient-relevant and reported outcome measures, particulay those related to pain on function, is integral to enhance generalizability. Including asymptomatic teeth at baseline helped investigate different types of postoperative pain without the confounding effect of preoperative pain. Future clinical trials with larger sample sizes, including patients with different maxillary and mandibular teeth types, and comparing piroxicam with other analgesic are recommended.

## Conclusions

A preoperative single oral dose of 20 mg sublingual fast-dissolving piroxicam can be effective in reducing spontaneous pain (POP) up to 24 h, stimulated pain (POPB and POPer) up to 7 days, and RAI incidence by at least 60% after single-visit endodontic treatment of asymptomatic mandibular molars with non-vital pulp; it can, also, inhibit any significant rise in POP and POPB postoperatively. Stimulated postoperative pain can be more severe and longer lasting than spontaneous pain.

## Supplementary Information

Supplementary Material 1.

## Data Availability

The data that support the findings of this study are available from the corresponding author upon reasonable requests.
